# Phototransformation of Graphene Oxide on the Removal of Sulfamethazine in a Water Environment

**DOI:** 10.3390/nano11082134

**Published:** 2021-08-22

**Authors:** Fei-Fei Liu, Meng-Ru Li, Su-Chun Wang, Yu-Xue Zhang, Guang-Zhou Liu, Jin-Lin Fan

**Affiliations:** 1 Institute of Marine Science and Technology, Shandong University, Qingdao 266237, China; limr666@163.com (M.-R.L.); suchunw@163.com (S.-C.W.); zhangyuxue@mail.sdu.edu.cn (Y.-X.Z.); liuguangzhou@sdu.edu.cn (G.-Z.L.); 2 Department of Science and Technology Management, Shandong University, Jinan 250100, China; fanjinlin@sdu.edu.cn

**Keywords:** graphene oxide, sulfamethazine, phototransformation, free radicals

## Abstract

Graphene oxide (GO) is widely used in various fields and has raised concerns regarding its potential environmental fate and effect. However, there are few studies on its influence on coexisting pollutants. In this study, the phototransformation of GO and coexisting sulfamethazine (SMZ) under UV irradiation was investigated, with a focus on the role of reactive oxygen species. The results demonstrated that GO promoted the degradation of SMZ under UV irradiation. The higher the concentration of GO, the higher the degradation rate of SMZ, and the faster the first-order reaction rate. Two main radicals, ∙OH and ^1^O_2_, both contributed greatly in terms of regulating the removal of SMZ. Cl^−^, SO_4_^2−^, and pH mainly promoted SMZ degradation by increasing the generation of ∙OH, while humic acid inhibited SMZ degradation due to the reduction of ∙OH. Moreover, after UV illumination, the GO suspension changed from light yellow to dark brown with increasing absorbance at a wavelength of 225 nm. Raman spectra revealed that the *I*_D_/*I*_G_ ratio slightly decreased, indicating that some of the functional groups on the surface of GO were removed under low-intensity UV illumination. This study revealed that GO plays important roles in the photochemical transformation of environmental pollutants, which is helpful for understanding the environmental behaviors and risks of nanoparticles in aquatic environments.

## 1. Introduction

As a kind of two-dimensional layered nanomaterial, graphene oxide (GO) possesses good mechanical, electrical, and thermal properties, and is widely applied in various fields, including biology, medicine, chemistry, and electronic engineering [1]. The global production of GO is expected to reach 3800 metric tons in 2027 [2]. Due to the presence of a large number of oxygen-containing functional groups, such as hydroxyl, carboxyl, and epoxy groups, GO has excellent hydrophilicity and a high probability of being present in natural aquatic environments, thus having uncertain environmental impacts and ecological risks. It has been reported that GO and its derivatives exhibited cytotoxicity to bacteria, biofilms, and algae [3,4]. Moreover, GO could cause developmental genotoxicity in aquatic animals such as zebrafish at trace concentrations [5], and could even accumulate in humans through the food chain [6]. Therefore, an increasing number of studies on the environmental behaviors of GO have received attention.

Once released into the environment, GO can interact with other pollutants mainly through π bonds, hydrophobic interactions, hydrogen bonds, and electrostatic interactions [7,8,9,10], thus affecting the transport and fate of coexisting compounds. For example, GO exhibited a high affinity for heavy-metal ions, which improved the transport ability of Pb^2+^ and Cd^2+^ in saturated porous media [11]. GO also facilitated the transport of antibiotics (levofloxacin, ciprofloxacin, and tetracycline) in saturated or unsaturated porous media because of the high sorption capacity of antibiotics by GO [12,13]. Furthermore, highly hydrophilic and mobile GO could serve as a carrier and promote the transport of nano-TiO_2_ in porous media [14]. In addition, the interaction between GO and other pollutants would change their combined toxicity to organisms. GO enhanced Cd toxicity on photosynthesis, biomass, and cell membrane lipids in wheat seedlings [15]. GO also promoted lipotoxicity and hepatic function deficits caused by *cis*-bifenthrin exposure in tadpoles [16]. Cao et al. revealed that environmentally relevant concentrations of GO (1 mg/L) significantly increased the phytotoxicity of As (III) and As (V) in plants, which resulted in more severe oxidative stress and a significant reduction in nutrient content [17].

However, it should be noted that GO may be subjected to the phototransformation process in the environment because its special sp^2^ domains can effectively adsorb sunlight, especially UV light [18,19,20]. GO was structurally degraded and chemically formed reduced GO under UV or sunlight irradiation [21]. After phototransformation, the toxicity of GO to bacteria (such as Gram-negative *Escherichia coli* and Gram-positive *Staphylococcus aureus*) and algal cells (*Chlorella pyrenoidosa*) was enhanced [22,23]. Meanwhile, GO can be regarded as a semiconductor with a zero energy gap to generate electrons, holes, and a series of reactive oxygen species (ROS) [24,25], which can mediate the transformation of the coexisting pollutants in the environment. For example, Cao et al. reported that silver nanoparticles could be formed from aqueous Ag^2+^ in the presence of GO under light [26]. Cu^2+^ on the surface of GO sheets could also trap e^−^ generated by GO and be reduced to Cu(Ⅰ) and then form Cu_2_O nanoparticles with the assistance of ROS, which suppressed the joint toxicity of GO and Cu^2+^ to freshwater algae after phototransformation [22]. In addition, GO could oxidize 42% of the adsorbed As (III) to As (V) under light irradiation, which was induced by electron-hole pairs on the surface of GO. However, coexposure to GO greatly enhanced the toxicity of As (III, V) to algae [27]. Therefore, it is of great significance to explore the photochemical transformation of GO on coexisting contaminants, especially when evaluating their environmental fate and possible toxicity and risks.

Antibiotics, as emerging contaminants, have gained increasing attention in recent years due to their widespread application and large production amounts [28,29]. As a result, antibiotics will inevitably find their way into the environment. Sulfamethazine (SMZ), one of the most common broad-spectrum antibiotics, is widely used in aquaculture, animal husbandry, hospitals, pharmaceutical factories, and other processes. Previous studies revealed that SMZ was frequently detected in wastewater, surface water, and even groundwater at concentrations ranging from ng L^−1^ to μg L^−1^ [30,31]. An increasing number of studies have focused on the environmental behaviors of SMZ, including its adsorption, migration, photooxidation, and so on [32,33,34,35].

Therefore, in this study, SMZ was selected as the model compound to reveal the effect of phototransformation of GO on coexisting contaminants. We systematically investigated the interaction between GO and SMZ under UV light, considering the influence of different environmental factors, including pH values, ionic strength and species, and natural organic matter (NOM). The phototransformation of GO together with the generation mechanisms of ROS were further explored to reveal the possible cotransformation pathways of antibiotics and GO.

## 2. Materials and Methods

### 2.1. Materials

GO was synthesized by an improved Hummers’ method [36]. SMZ (≥99%) was purchased from Aladdin Biochemical Technology Co., Ltd. (Shanghai, China). The other reagents used in this study were obtained from Sinopharm Chemical Reagent Co., Ltd. (Shanghai, China). All aqueous samples were prepared with ultrawater.

### 2.2. Photochemistry Experiment

All experiments were conducted in a multichannel photocatalytic reaction system (PCX50C, Beijing Perfect Light Science and Technology Co., Ltd., Beijing, China). The system was operated at an average light intensity of 10.0 mW cm^−2^ with 5 W LED white lamps (365 nm). During the 6 h photochemical experiments, 50 mL of the reaction solutions was magnetically stirred at 300 rpm in quartz tubes that were maintained at constant temperature (22 ± 2 °C) with the circulating water bath of the reactor.

### 2.3. SMZ Degradation

One batch experiment was first conducted with a fixed amount of SMZ (5 μM) with GO ranging from 10 mg/L to 50 mg/L. Dark control experiments were also conducted under the same conditions. To investigate the effects of solution chemistry factors on the photochemical transformation, another three sets of experiments were also performed with 5 μM SMZ and 30 mg/L GO. The pH effect experiments were conducted with the solution pH ranging from 3.0 to 9.0, which was adjusted with 0.1 M HCl or NaOH. Ionic strength and species effect experiments were performed in the presence of 0–600 mM NaCl or 0–30 mM Na_2_SO_4_. In addition, the photochemical transformation of SMZ was tested in the presence of humic acid (HA) in the range of 0–10 mg/L. All the above experiments were performed in triplicate. During the experiments, 3 mL of solution was sampled at determined time intervals and filtered with 0.22 μm nylon membranes to remove GO. Then, SMZ was analyzed at a determination wavelength of 270 nm by high-performance liquid chromatography (HPLC, Shimadzu LC-20AT, Tokyo, Japan) with a UV detector using a C18 column (25 cm × 4.6 mm, 5 μm). The mobile phase was acetonitrile/0.05 M acetic acid (30:70, *v:v*) with a flow rate of 1 mL/min. The injection volume was 10 μL, and the column temperature was maintained at 40 °C.

### 2.4. ROS Generation

It should be noted that O_2_∙^−^ was not detected with the XTT sodium salt (probe for O_2_∙^−^) in this study; therefore, we only focused on the production of ∙OH and ^1^O_2_. Free radical quenching experiments were first carried out with L-histidine and potassium iodide (KI) as radical quenchers to identify the contribution of ^1^O_2_ and ∙OH, respectively [37,38]. The inhibition rate of SMZ degradation was determined after introducing free radical scavengers. In addition, 200 μM terephthalic acid (TPA) and 300 μM furfuryl alcohol (FFA) were used as indicators to quantify the amount of ROS [39,40]. TPA reacted with ∙OH and produced 2-hydroxyterephthalic acid (HTPA), which could be measured by a fluorescence spectrophotometer (HITACHI, F-2500, Tokyo, Japan). The excitation and emission wavelengths were 315 nm and 425 nm, respectively [23,40]. FFA was analyzed by HPLC at 218 nm. The mobile phase was 30% acetonitrile and 70% phosphoric acid and run at 1.0 mL/min.

### 2.5. GO Characterization

To investigate the phototransformation of GO, the changes in GO in the photoreaction system were characterized by UV–vis spectrophotometry from 200 nm to 600 nm. Additionally, the Raman spectra were measured at 1000 cm^−1^ to 2000 cm^−1^ with 532 nm excitation with a Raman spectrometer (Renishaw inVia Reflex, New Mills, UK) before and after UV illumination.

## 3. Results and Discussion

### 3.1. SMZ Degradation

**Figure 1:**
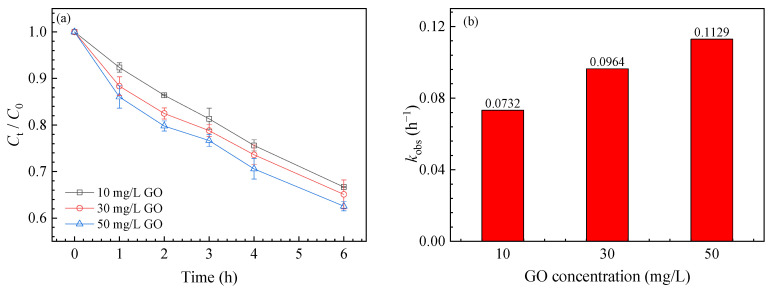
Effects of GO on SMZ degradation under UV light (**a**) and *k*_obs_ (**b**).

The effects of different concentrations of GO on SMZ degradation were first studied. GO did not adsorb SMZ much in the dark, and little degradation of SMZ occurred under UV illumination without GO (Figure S1). However, the degradation of SMZ was accelerated in the presence of GO. The degradation rates of SMZ were 33.32 ± 2.54%, 34.90 ± 2.69%, and 37.44 ± 2.12% in the presence of 10 mg/L, 30 mg/L, and 50 mg/L GO, respectively (Figure 1a). According to the first-order kinetic fitting of the reaction in the first two hours (Figure S2), the observed reaction rate constants (*k*_obs_) were 0.0732 h^−1^, 0.0964 h^−1^, and 0.1129 h^−1^ (Figure 1b).

### 3.2. ROS Generation

**Figure 2:**
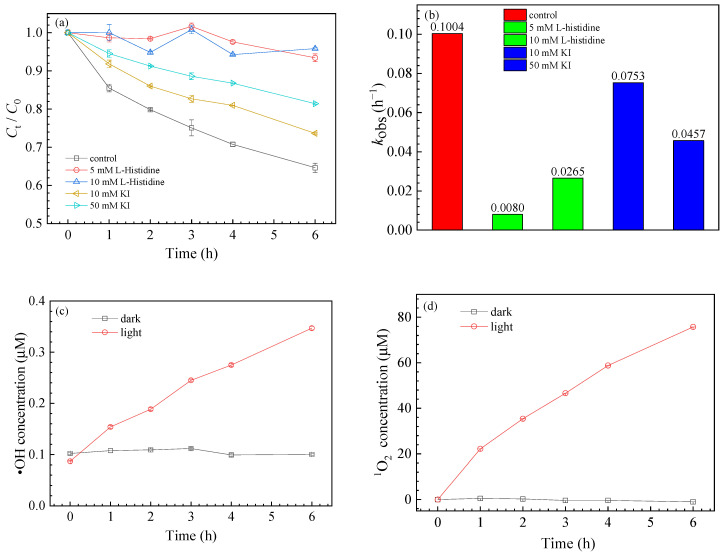
Degradation kinetics of SMZ (**a**) and *k*_obs_ (**b**) with free radical scavengers: L-histidine and KI; generation kinetics of ∙OH (**c**) and ^1^O_2_ (**d**).

Generally, nanoparticles can generate ROS under UV light irradiation, which can participate in the degradation of chemicals. ROS generation by GO is similar to that of semiconductors. A large number of oxygen-containing functional groups attached to the GO surface play an important role in electron transfer and promote ROS generation [21]. To further explore the mechanism of GO on SMZ transformation, free radical scavengers, including L-histidine and KI, were added to the reaction solution to identify the role of ^1^O_2_ and ∙OH. As shown in Figure 2a,b, 5 mM/10 mM L-histidine significantly inhibited SMZ degradation, reducing its degradation rate from 32.52 ± 4.34% to 6.57 ± 3.24% and 4.18 ± 1.63%, with *k*_obs_ decreasing from 0.1004 h^−1^ to 0.0080 h^−1^ and 0.0265 h^−1^ (Figure S3), respectively. Similar results were also observed in the presence of KI, where the decomposition of SMZ reduced to 26.36% for 10 mM KI and 18.59% for 50 mM KI. Compared with the initial *k*_obs_ of 0.1004 h^−1^, *k*_obs_ decreased to only 0.0753 h^−1^ and 0.0457 h^−1^ (Figure S3), respectively. Thus, the above results showed that both ^1^O_2_ and ∙OH participated in SMZ degradation.

ROS quantification was performed during the photochemical experiments. Figure 2c,d shows that the free radical production of GO was proportional to the illumination time; 75.70 μM ^1^O_2_ and 0.35 μM ∙OH could be produced after 6 h of illumination in the presence of 30 mg/L GO. Based on the above experimental results, possible ROS generation pathways were further proposed, as shown in the following reaction formulas [23,41,42]:

(1) $$\mathrm{GO}+\mathrm{hv}\to {\mathrm{GO}}^{*}\;\left(e_{\mathrm{CB}}^{-}-h_{\mathrm{VB}}^{+}\right),$$

(2) $${\mathrm{GO}}^{*}+O_{2}\to O_{2}{}^{1}+\mathrm{GO},$$

(3) $$e_{\mathrm{CB}}^{-}+O_{2}\to O_{2}\cdot^{-},$$

(4) $$h_{\mathrm{VB}}^{+}+H_{2}O\to \cdot \mathrm{OH}+H^{+},$$

(5) $$h_{\mathrm{VB}}^{+}+{\mathrm{OH}}^{-}\to \cdot \mathrm{OH},$$

(6) $$O_{2}\cdot^{-}+h_{\mathrm{VB}}^{+}\to O_{2}{}^{1},$$

(7) $$O_{2}\cdot^{-}+e_{\mathrm{VB}}^{-}+2H^{+}\to H_{2}O_{2},$$

(8) $$H_{2}O_{2}+e_{\mathrm{CB}}^{-}\to \cdot \mathrm{OH}+{\mathrm{OH}}^{-},$$

### 3.3. Effects of Different Conditions on SMZ Degradation

#### 3.3.1. Effect of pH

**Figure 3:**
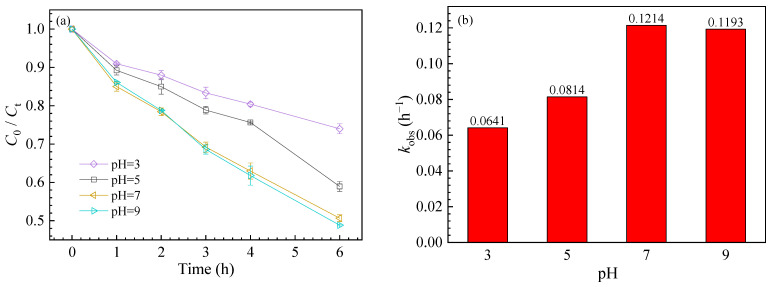
Effect of pH on SMZ degradation kinetics (**a**) and *k*_obs_ of SMZ degradation (**b**).

The pH value of the solution has a great influence on the photolysis of SMZ (Figure 3). The degradation rates were 26.02 ± 3.05%, 41.06 ± 4.23%, 49.33 ± 5.11%, and 51.14 ± 5.63% as the pH increased from 3.0 to 9.0, and *k*_obs_ were 0.0641 h^−1^, 0.0814 h^−1^, 0.1214 h^−1^, and 0.1193 h^−1^ (Figure S4), respectively. The relative high degradation of SMZ at higher pH conditions was probably due to the following two reasons: firstly, SMZ (*p*K_a1_ = 2.6, *p*K_a2_ = 8.0) can be degraded more easily in its ionic forms compared with the neutral form [43]. Secondly, similar to semiconductors [44], GO produces holes after UV illumination, which can further react with OH^−^ to produce ∙OH [45,46]. The generation of ∙OH increased with increasing pH, resulting in the promotion of SMZ conversion. In addition, the dispersion of GO was higher at higher pH because of the deprotonation of oxygen-containing functional groups on the GO surface [47], which might result in an increase in the steady-state concentration of ROS. Therefore, SMZ degradation by GO was higher at high pH than at low pH.

#### 3.3.2. Effect of Coexisting Anions

**Figure 4:**
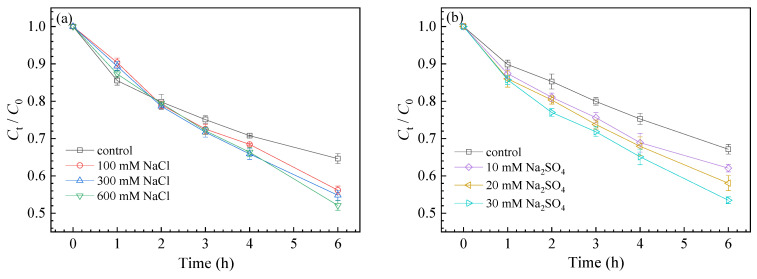
Effect of Cl^−^ (**a**) and SO_4_^2−^ (**b**) on SMZ degradation kinetics.

The effects of ionic strength and species on SMZ degradation are presented in Figure 4. NaCl improved the degradation of SMZ, with the degradation rate rising from 35.36 ± 1.69% to 43.83 ± 2.21%, 45.18 ± 2.88%, and 47.9 ± 2.79% in the presence of NaCl from 100 mM to 600 mM, respectively. Similarly, when 10 mM, 20 mM, and 30 mM Na_2_SO_4_ were added to the solution, the SMZ decomposition rate increased to 37.92 ± 2.38%, 41.94 ± 2.57%, and 46.52± 2.78%, respectively.

**Figure 5:**
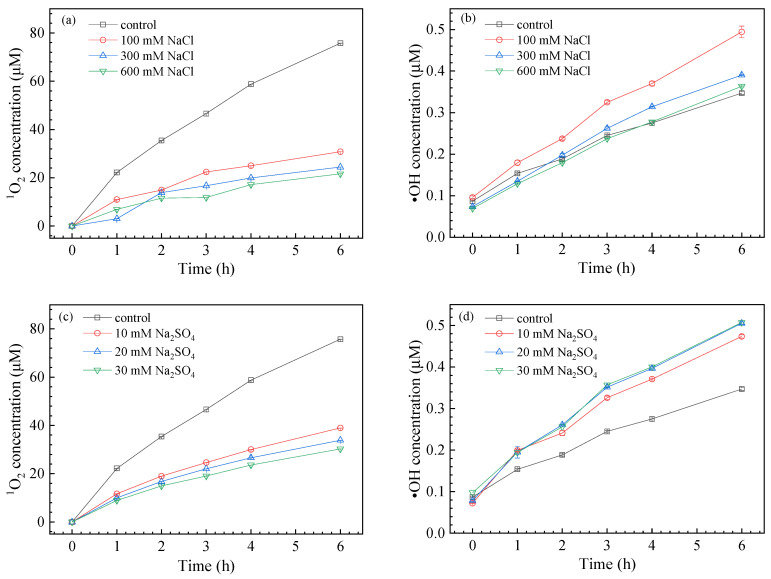
Effects of Cl^−^ and SO_4_^2−^ on ^1^O_2_ production (**a**,**c**) and ∙OH production (**b**,**d**).

To further explore the effect of Cl^−^ and SO_4_^2−^ on the photolysis of SMZ, quantitative analysis of ^1^O_2_ and ∙OH was carried out (Figure 5). It was evident that Cl^−^ showed a negative influence on the production of ^1^O_2_, whose level was reduced to 21.63 μM with increasing Cl^−^ concentration, compared with that of the control 75.70 μM. However, the presence of Cl^−^ accelerated the generation of ∙OH, especially 100 mM NaCl, which increased the amount of ∙OH by 1.6 times compared with the control. This could be explained by the fact that Cl^−^ generated hydrated electrons under UV irradiation, which were then transferred to nanomaterials to generate more ROS (Equation (9)) [48]. It should be noted that excessive Cl^−^ would agglomerate GO under high ionic strength [48,49], which would reduce the surface area of GO and the concentration of ROS. Thus, the steady-state concentration of ∙OH first increased and then decreased with increasing NaCl concentration. The presence of Cl^−^ promoted the decomposition of SMZ, which was in accordance with the role of ∙OH. Therefore, ∙OH was expected to be the main ROS species that regulated SMZ degradation.

(9) $${\mathrm{Cl}}^{-}+\mathrm{hv}\to \mathrm{Cl}+e_{\mathrm{aq}}{}^{-},$$

Similar to Cl^−^, the presence of SO_4_^2−^ also inhibited the generation of ^1^O_2_ but prompted the production of ∙OH. The concentration of ^1^O_2_ decreased from 75.70 μM to 30.28 μM with increasing SO_4_^2−^ from 0 to 30 mM, but the ∙OH concentration gradually increased from 0.35 μM to 0.51 μM. Therefore, the introduction of SO_4_^2−^ into the solution promoted SMZ degradation by increasing the steady-state concentration of ∙OH. On the other hand, SO_4_^2−^ existing on the GO surface would form reactive sulfate radicals by holes (Equation (10)) [46], which may also accelerate the transformation of SMZ [50].

(10) $${\mathrm{SO}}_{4}^{2-}+h^{+}\to {\mathrm{SO}}_{4}^{.-},$$

#### 3.3.3. Effect of NOM

**Figure 6:**
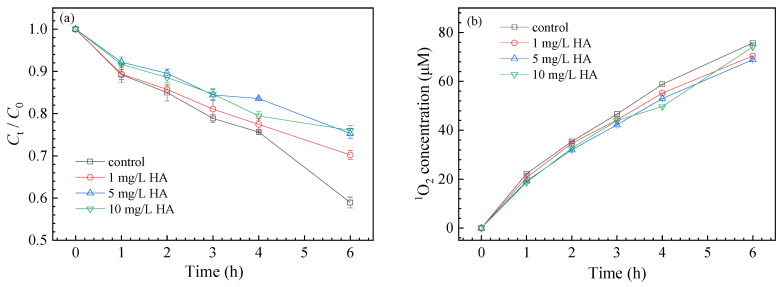
Effects of HA on SMZ degradation kinetics (**a**) and ^1^O_2_ production (**b**).

As the representative NOM, HA is a macromolecular polymer containing carboxyl, phenolic, and keto groups, which is widely distributed in natural waters. Previous studies showed that NOM might play different roles in the transformation of organic pollutants. For example, Chen et al. reported that HA could consume a large amount of ∙OH under UV light [51], which decreased the degradation of diethyl phthalate. However, Niu et al. proposed that NOM could be transformed into excited-state substances or free radicals under UV irradiation, which enhanced the degradation of norfloxacin [38]. In the present study, as shown in Figure 6, the degradation of SMZ significantly decreased from 41.06 ± 2.34%, to 29.80 ± 2.64%, 24.72 ± 2.56%, and 23.95 ± 2.59% in the presence of 1 mg/L, 5 mg/L and 10 mg/L HA, respectively. HA had an inhibitory effect on the degradation of SMZ. As HA might react with ∙OH, we only measured the production of ^1^O_2_ in the presence of HA. As shown in Figure 6b, compared with the control, HA slightly influenced the generation of ^1^O_2_, indicating that ^1^O_2_ contributed little to SMZ degradation. Therefore, it could be speculated that HA mainly quenched ∙OH to decrease the decomposition of SMZ.

### 3.4. GO Transformation

Under UV illumination, the color of the GO suspension changed from light yellow to dark brown (Figure S5), indicating that some oxygen-containing functional groups attached to the GO surface might be removed [19]. The variation of the solution absorbance with time was further determined by UV–vis spectrophotometry (Figure S6). The peak at 225 nm was attributed to the π -π* transition of unsaturated C-C bonds of GO. After 6 h of UV irradiation, the absorbance at 225 nm increased, indicating that the sp^3^ structure of GO was reduced and the sp^2^ structure had been recovered [21]. It should be noted that our previous study demonstrated that UV light intensity greatly affected the absorbance of GO, and the absorption peak at 225 nm could be redshifted to 255 nm at a high light intensity of 54 mW cm^−2^ in 4 h [23]. In the present study, the light intensity was only 10 mW cm^−2^; thus, the absorption peak did not shift significantly. Raman spectra were further used to analyze the GO samples before and after UV illumination. The D band at approximately 1350 cm^−1^ and the G band at approximately 1580 cm^−1^ are the two characteristic peaks of GO. Peak D represents the vibration of sp^3^ carbon atoms, and peak G is the characteristic peak of carbon sp^2^. The ratio of *I*_D_/*I*_G_ is usually used as a qualitative measurement of the disorder degree caused by nonaromatic sp^3^ carbon defects. After 6 h of illumination, *I*_D_/*I*_G_ decreased only from 0.8481 to 0.8438 (Figure S7), indicating that the graphitization structure of GO was somewhat improved and that the sp^2^ region was expanded. However, the insignificant decrease in *I*_D_/*I*_G_ suggested that the UV light intensity was not high enough in the current study, which was in accordance with the changes in UV–vis absorbance.

**Figure 7:**
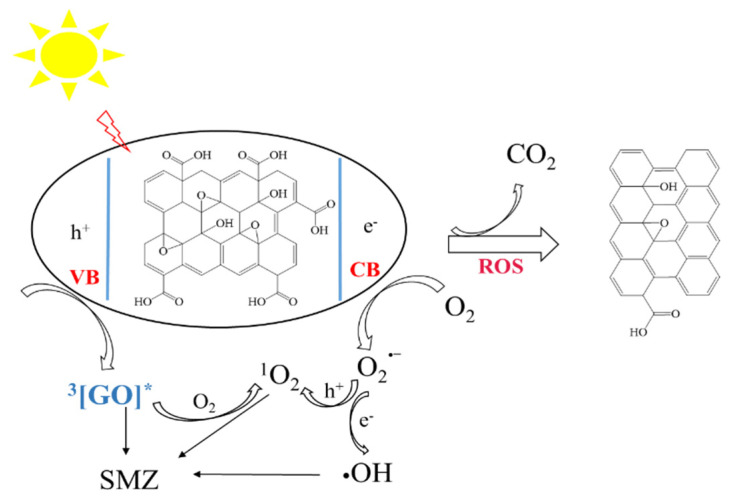
Proposed pathways for ROS generation and transformation of GO and SMZ under UV light.

Based on the above results, we proposed the possible cotransformation pathways of GO and SMZ (Figure 7). Similar to semiconductors, GO generated electrons and holes under illumination. Electrons could be captured by O_2_ to generate O_2_∙^−^, which was further converted into ^1^O_2_ and ∙OH. Meanwhile, GO could form excited-state GO* under illumination, and then O_2_ accepted excess energy and generated ^1^O_2_. Therefore, ROS generated in the above ways promoted SMZ degradation. At the same time, GO could capture electrons to reduce its surface oxygen-containing functional groups.

## 4. Conclusions

In this study, the photochemical behaviors of GO and the degradation of SMZ were quantitatively analyzed. GO could promote the degradation of SMZ under UV light. ∙OH and ^1^O_2_ were the main free radicals participating in the cotransformation between GO and SMZ. High pH, Cl^−^, and SO_4_^2^^−^ improved the degradation of SMZ by affecting the formation of ∙OH. However, the presence of HA consumed ∙OH, leading to less degradation of SMZ. As for GO, its color changed from light yellow to dark brown under UV illumination. However, the absorption peak did not shift significantly, and the ratio of *I*_D_/*I*_G_ was slightly smaller, which indicated that GO was somewhat reduced. The findings of this work may have significant implications for predicting the fate and assessing the potential risks of environmental pollutants and nanoparticles. However, to better understand the environmental behaviors of nanoparticles, long-term experiments under natural solar radiation are still needed.

## Data Availability

The datasets generated during and/or analyzed during the current study are available from the corresponding author on reasonable request.

## Supplementary Materials

The following are available online at https://www.mdpi.com/article/10.3390/nano11082134/s1, Figure S1: Figure S1. Photolysis kinetics of SMZ (5 μM) under UV light without GO (a) and the adsorption of SMZ (5 μM) by GO (10 and 100 mg/L) in the dark within 6 h (b), Figure S2: Pseudo first-order fitting results for SMZ degradation kinetics under various GO concentrations (10-50 mg/L), Figure S3: Pseudo first-order kinetics fitting for kinetics of SMZ degradation with L-histidine and KI, Figure S4: Pseudo first-order kinetics fitting for kinetics of SMZ degradation at pH 3.0-9.0, Figure S5: Changes in the color of GO under UV light as a function of irradiation time, Figure S6: Variation of GO absorbance with time under light, Figure S7: Raman spectra of GO before and after UV illumination.
