# Supplementary material for: Phototransformation of Graphene Oxide on the Removal of Sulfamethazine in a Water Environment
Source: Nanomaterials (Basel). 2021 Aug 22;11(8):2134. doi: 10.3390/nano11082134 (PMC8398241; doi:10.3390/nano11082134)
Supplement: Supplementary file 1 [file nanomaterials-11-02134-s001.zip › nanomaterials-1321664-SI.pdf]

nanomaterials

MDPI

Article

Phototransformation of Graphene Oxide on the Removal of Sulfamethazine in a Water Environment

Fei-Fei Liu [1,*], Meng-Ru Li [1], Su-Chun Wang [1], Yu-Xue Zhang [1], Guang-Zhou Liu [1] and Jin-Lin Fan [2]

[1] Institute of Marine Science and Technology, Shandong University, Qingdao 266237, China; limr666@163.com (M.-R.L.); suchunw@163.com (S.-C.W.); zhangyuxue@mail.sdu.edu.cn (Y.-X.Z.); liuguangzhou@sdu.edu.cn (G.-Z.L.)
[2] Department of Science and Technology Management, Shandong University, Jinan 250100, China; fanjinlin@sdu.edu.cn
* Correspondence: liufeifei@sdu.edu.cn

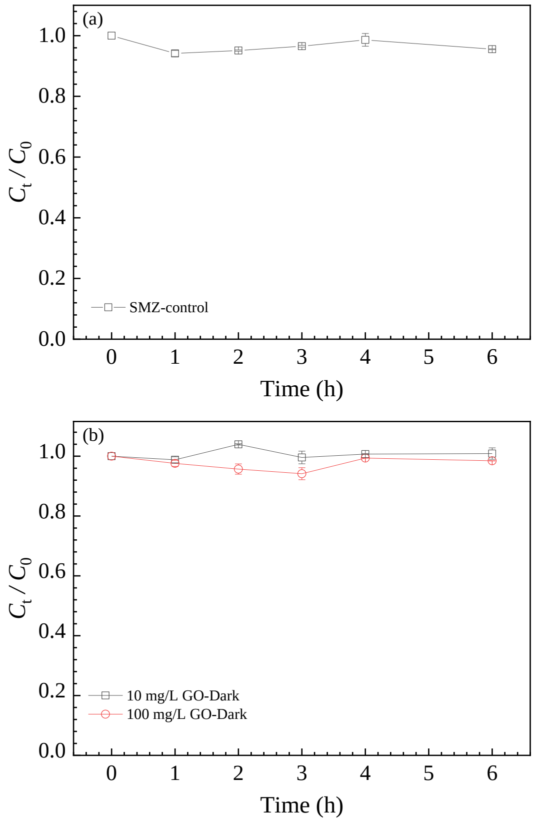

Figure S1. Photolysis kinetics of SMZ (5 μM) under UV light without GO (**a**) and the adsorption of SMZ (5 μM) by GO (10 and 100 mg/L) in the dark within 6 h (**b**).

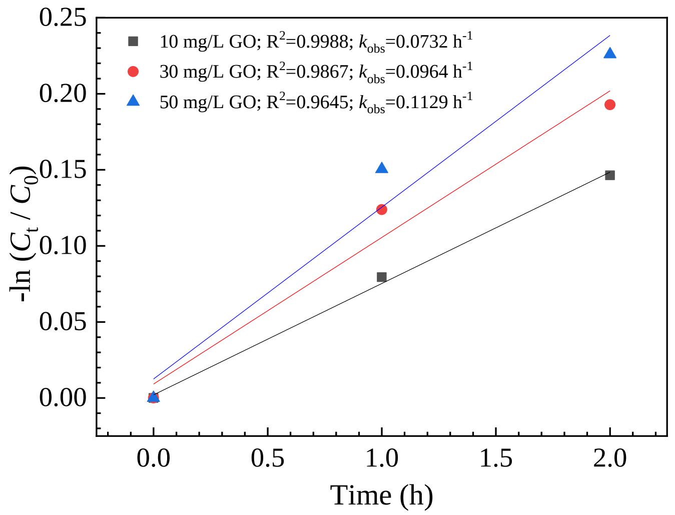

Figure S2. Pseudo first-order fitting results for SMZ degradation kinetics under various GO concentrations (10-50 mg/L).

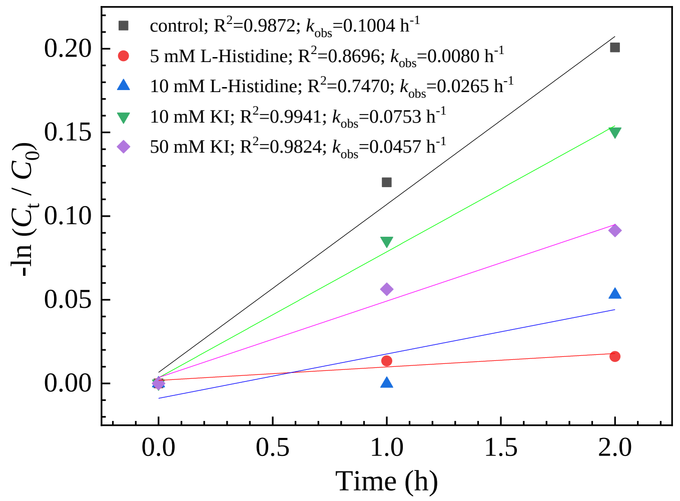

Figure S3. Pseudo first-order kinetics fitting for kinetics of SMZ degradation with L-histidine and KI.

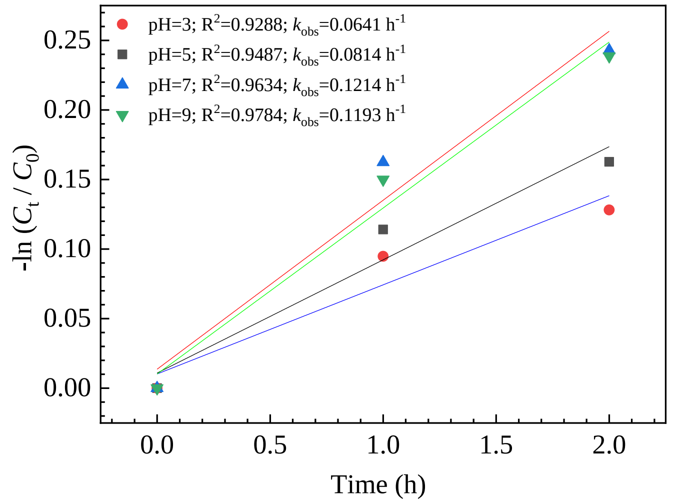

Figure S4. Pseudo first-order kinetics fitting for kinetics of SMZ degradation at pH 3.0-9.0.

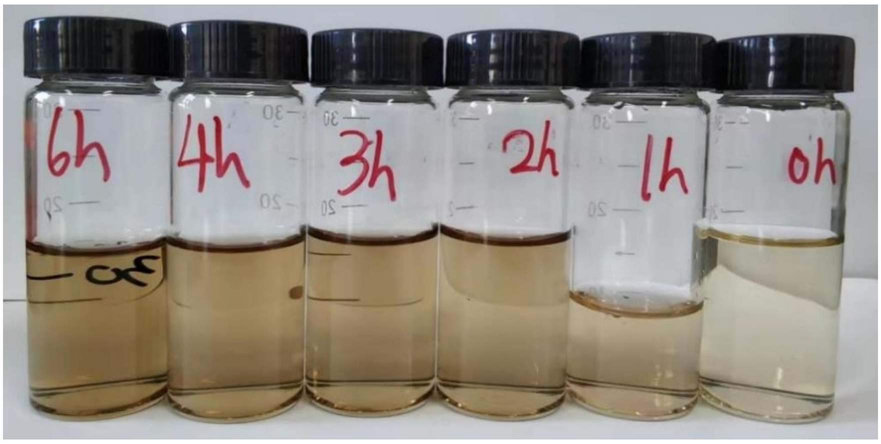

Figure S5. Changes in the color of GO under UV light as a function of irradiation time.

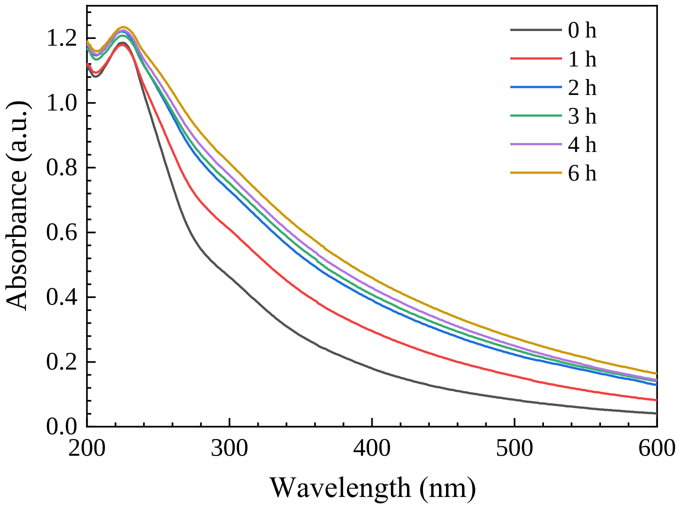

Figure S6. Variation of GO absorbance with time under light.

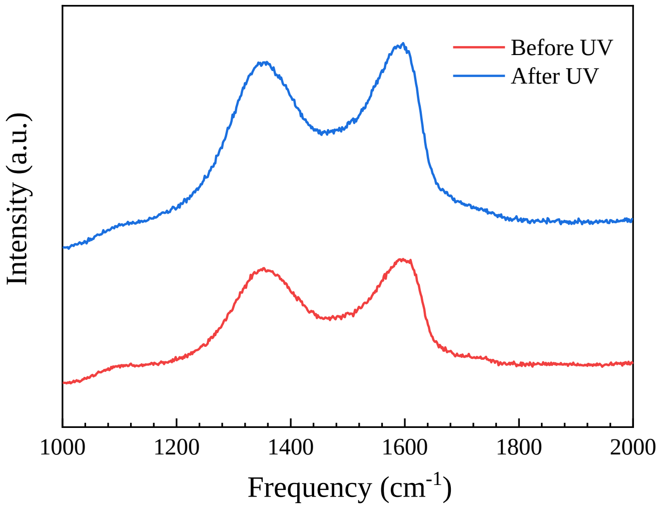

Figure S7. Raman spectra of GO before and after UV illumination.
